# Endoscopic submucosal dissection under general anesthesia for superficial esophageal squamous cell carcinoma is associated with better clinical outcomes

**DOI:** 10.1186/s12876-018-0813-z

**Published:** 2018-06-07

**Authors:** Byeong Geun Song, Yang Won Min, Ra Ri Cha, Hyuk Lee, Byung-Hoon Min, Jun Haeng Lee, Poong-Lyul Rhee, Jae J. Kim

**Affiliations:** Department of Medicine, Samsung Medical Center, Sungkyunkwan University School of Medicine, 81 Irwon-ro, Gangnam-gu, Seoul, 06351 South Korea

**Keywords:** Endoscopic submucosal dissection, Esophageal squamous cell carcinoma, General anesthesia, Sedation, Outcome

## Abstract

**Background:**

Endoscopic submucosal dissection (ESD) has been widely accepted for treating superficial esophageal squamous cell carcinoma (SESCC). The aim of this study was to evaluate the efficacy and safety of ESD for SESCC and the effect of different sedation methods on their clinical outcomes.

**Methods:**

We retrospectively analyzed a total of 169 patients (175 lesions) who underwent ESD for SESCC at Samsung Medical Center, Seoul, South Korea. Short-term and long-term clinical outcomes were evaluated and compared according to the sedation method (conscious sedation [CS] vs general anesthesia [GA]).

**Results:**

En bloc resection, complete resection, and curative resection (CuR) were achieved in 93.7, 74.9, and 58.9% of cancers, respectively. Perforation and stricture occurred in 8.0 and 12.0% of lesions, respectively. During a mean follow-up period of 33.7 months for survival, 3 (3.0%) patients died without evidence of recurrence after achieving CuR. During a mean follow-up period of 32.5 months for recurrence, 1 (1.0%) patient experienced lymph node metastasis. Synchronous and metachronous cancer were found in 1.0% and in 3.0% of patients, respectively. Multivariate analysis revealed that GA was associated with a higher complete resection rate and a lower perforation rate as compared to CS (odds ratio 3.401, 95% confidence interval 1.317–8.785, *P* = 0.011 and odds ratio 0.067, 95% confidence interval 0.006–0.775, *P* = 0.030, respectively).

**Conclusions:**

ESD is an oncologically effective treatment modality for SESCC, particularly when CuR is achieved. Applying GA for esophageal ESD could improve the clinical outcomes of ESD in patients with SESCC.

## Background

Esophageal cancer was ranked the ninth for cancer incidence and the sixth for cancer death in 2013 [1]. Due to aging and growing population, esophageal cancer cases have increased compared to that in 1990. The majority of esophageal cancers are squamous cell carcinomas (SCCs) in the Middle East, Africa, Asia, and parts of Europe [2, 3]. Given the considerable morbidity and mortality of esophagectomy [4–6], endoscopic submucosal dissection (ESD) has been used for superficial esophageal SCC (SESCC) without metastasis [7]. Furthermore, SESCC is frequently detected due to nationwide screening endoscopy for gastric cancer together with the development of advanced diagnostic techniques such as image-enhanced endoscopy in East Asia including Korea [8–11].

Safety and efficacy of ESD for early gastric cancer (EGC) have been sufficiently verified in many studies [12–14]. However, SESCC is different from EGC when considered for ESD. SESCC has a relatively higher risk of lymph node metastasis (LNM) even when it is confined to the mucosa [15, 16]. The risk of complications such as stricture and perforation is also higher in SESCC [17–19]. However, there is nonsurgical treatment option such as radiotherapy with or without chemotherapy after non-curative ESD for SESCC [20, 21]. Although several studies have reported the outcomes of ESD for SESCC, most of them were of small sample size and were performed in Japanese population [17, 18, 22–27]. Thus, there is still a need for further outcome data to be reported.

Given the relatively higher complication risk of esophageal ESD, ESD for SESCC is frequently performed under general anesthesia (GA). Indeed, a recent study reported that there was no perforation in 58 esophageal ESDs performed under GA [28]. However, till date, there has been no comparison between GA and conscious sedation (CS) in this aspect.

The aims of this study were (i) to evaluate the clinical outcomes of ESD for SESCC and (ii) to compare the short-term outcomes between ESD under CS and that under GA.

## Methods

### Patients

**Fig. 1:**
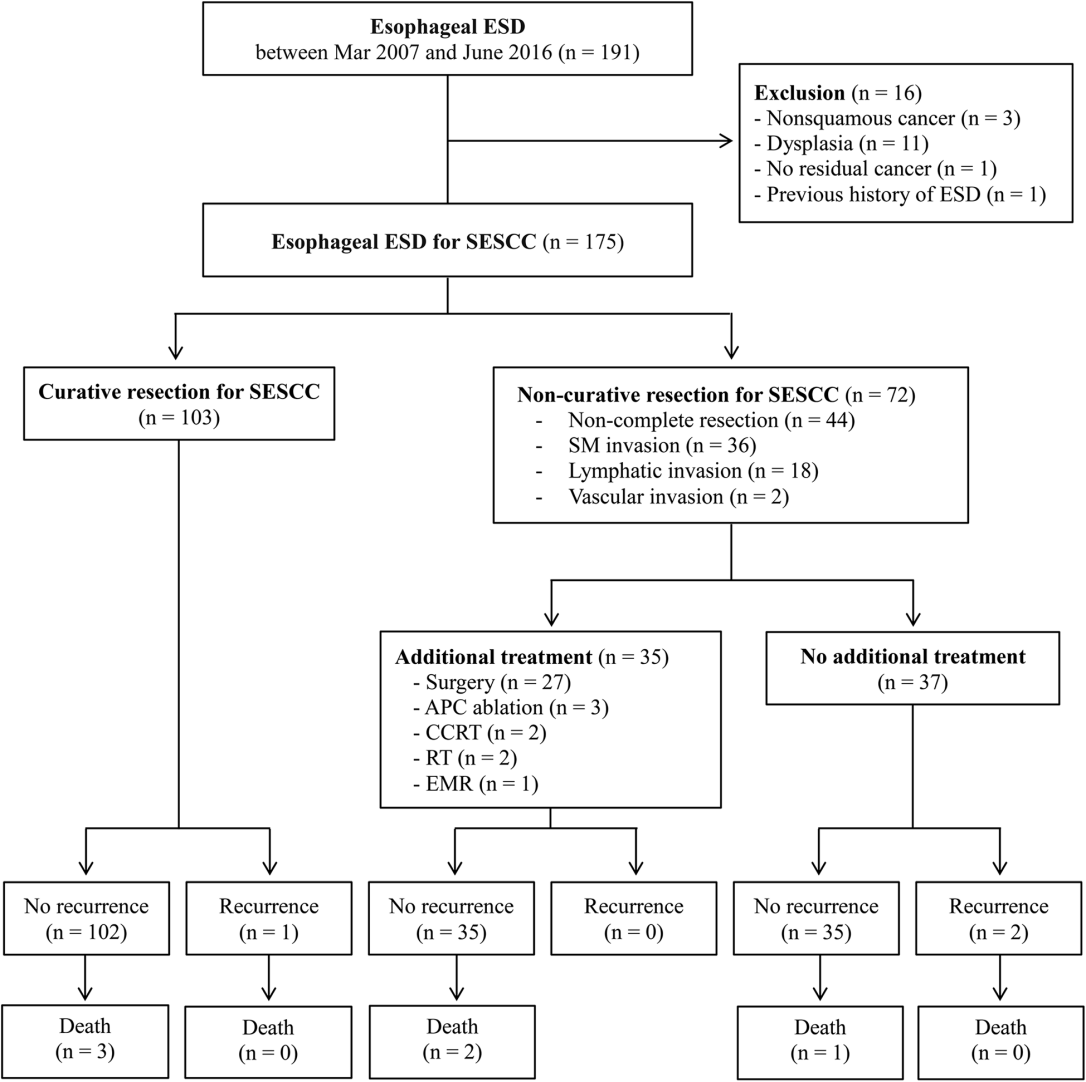
Patient flow diagram of this study. ESD, endoscopic submucosal dissection; SESCC, superficial esophageal squamous cell carcinoma; SM, submucosal; APC, Argon plasma coagulation; CCRT, concurrent chemoradiotherapy; RT, radiotherapy; EMR, endoscopic mucosal resection

Patients who underwent ESD for SESCC at Samsung Medical Center, Seoul, South Korea, between March 2007 and June 2016 were eligible. Based on the final pathologic report, only SCC cases were included. All patients underwent endoscopic evaluation including chromoendoscopy with the Lugol’s iodine dye-spraying method. Most of them also underwent endoscopic ultrasound (EUS) to confirm that the cancer was confined to the mucosa except those patients who had mucosal cancer confirmed by EUS which were performed prior to being referred to our institution. In addition, computed tomography (CT) scans of the chest and abdomen were performed in all patients prior to ESD to identify possible distant or local LNM. Finally, esophageal ESD was only performed for those SESCC that were confined to the mucosal layer without distant or local LNM excluding those with obvious SM invasion (Fig. 1). This study was conducted in accordance with the Declaration of Helsinki and approved by the Institutional Review Board at Samsung Medical Center (No. 2016–08-192).

### Procedures and follow-ups

**Fig. 2:**
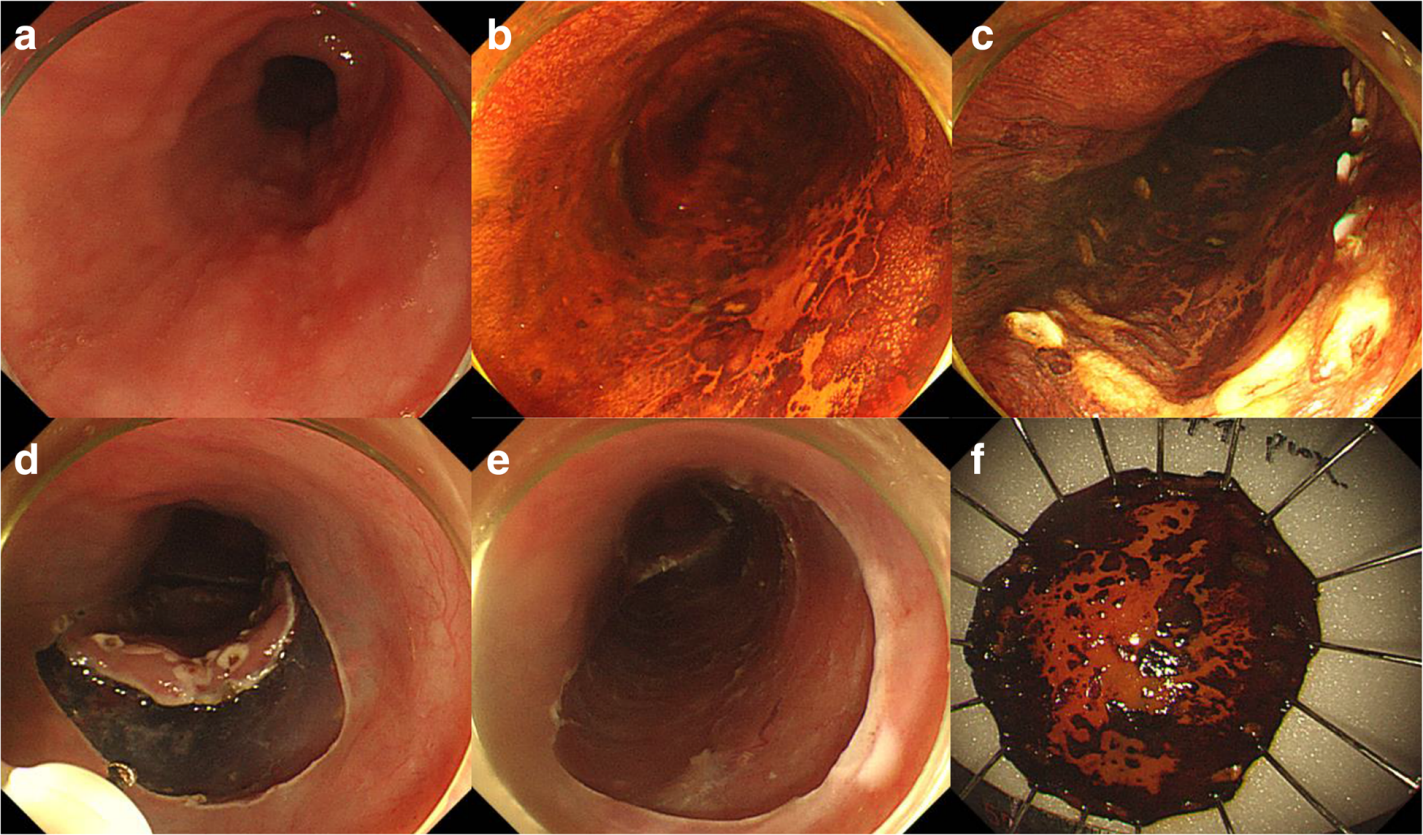
Endoscopic submucosal dissection (ESD) procedure. **a** On the mid esophagus, a 2.6-cm-sized geographic mucosal hyperemia is noticed. **b** Lugol’s solution is sprayed along the lesion to aid visualization. **c** Marking around the lesion is performed. **d** After submucosal injection, circumferential mucosal pre-cutting is performed. **e** After dissection of the submucosal layer, an artificial ulcer is seen. **f** Fixation of the resected specimen

Six endoscopists performed esophageal ESD using standard technique as described elsewhere [29, 30]. At first, marking around the cancer is done 2–3 mm away from the edge which is well determined by Lugol’s iodine chromoendoscopy. Then circumferential mucosal pre-cutting is performed after submucosal injection. After elevating the lesion by submucosal injection, submucosal layer under the lesion is dissected using various types of ESD knives (Fig. 2).

In the initial phase of the study period, esophageal ESD was mainly performed under CS. For sedation under CS, midazolam and pethidine were administered intravenously by the endoscopist. Since August 2012, selected cases were sedated using intravenous midazolam, propofol, and remifentanil by the anesthesiologist. Since March 2014, when anesthesia machine became available in the endoscopy room, almost all esophageal ESD were performed under GA with endotracheal intubation by the anesthesiologist. GA is induced with rocuronium, midazolam, and propofol and is maintained with propofol and remifentanil.

Six endoscopists performed esophageal ESD in the present study. Among them, three performed ESD under CS, and their mean experience of esophageal ESD was 23.7 cases. The other three performed ESD under CS or GA, and their mean experience was 34.7 cases.

Patients without any complications were discharged from the hospital at day 4 after ESD. To prevent post-ESD stricture, polyglycolic acid sheet application [31] or oral prednisolone administration [32, 33] was applied in patients with a large mucosal defect at the discretion of the endoscopist. Oral prednisolone administration was usually started at a dose of 30 or 40 mg/day, tapered gradually, and then discontinued after 8 weeks.

After curative ESD, upper endoscopy was performed at two months after ESD to exclude the presence of any residual tumor. Endoscopy and chest and abdomen CT scans were performed every 6 months for 3 years. From the 4th to the 5th year after ESD, endoscopy and CT scans were performed annually.

### Data collection and definitions

Data in this study were obtained from a prospectively collected database of esophageal ESD. These data included demographics parameters (such as patient’s age, gender, past medical history, and body mass index), tumor characteristics (such as the tumor location, endoscopic tumor morphology, gross and pathologic size of the tumor, pathology of the tumor, circumferential size of the tumor and post-ESD mucosal defect), and procedure-related factors (procedure time, method of sedation, and complications).

Tumor location was described according to the American Joint Committee on Cancer divisions of the esophagus [34]. Upper thoracic esophagus refers to the segment of esophagus at a distance of 20 cm to 25 cm from the incisor while middle thoracic esophagus, 25 cm to 30 cm and lower thoracic esophagus, 30 cm to typically, 40 cm. Esophagogastric junction refers to the region between the terminal end of the esophagus and beginning of the stomach at the cardiac orifice.

### Assessments

Data were analyzed with respect to the en bloc resection (EnR) rate, complete resection (CR) rate, curative resection (CuR) rate, death rate, recurrence rate (local or distal recurrence), presence of synchronous and metachronous cancer, complications, procedure time, and length of hospital stay.

Resected specimens were fixed in formalin and serially sectioned perpendicularly at 2 mm intervals. Depth of invasion was classified into M1 (confined to the intraepithelium), M2 (confined to the lamina propria), M3 (confined to the muscularis mucosa), SM1 (submucosal invasion < 200 μm), and SM2&3 (submucosal invasion ≥200 μm) [35]. EnR was defined as resection of targeted lesions in one piece regardless of the depth of invasion or lymphovascular invasion (LVI). CR was defined as EnR with histologically confirmed tumor-negative margins. CuR was defined as CR without SM invasion, LVI, or poorly differentiated histology. Non-CuR was defined as tumors that did not fulfill the above criteria for CuR.

Local recurrence was defined as a histologically confirmed recurred SCC at the site of ESD after an initial CR. Distant recurrence was defined when a new malignant lesion was detected outside of the esophagus. Synchronous cancer was defined as a histologically confirmed recurred cancer at a different location from the ESD site within 1 year after ESD. Metachronous cancer was defined when recurred cancer was detected more than 1 year after ESD. Procedure time was defined as the interval of time between the first marking and the completion of submucosal dissection.

### Complications

The incidence of ESD-related complications including perforation (micro-perforation and frank perforation), bleeding, and stricture were estimated per lesion. Micro-perforation was defined as radiographic evidence of free air or subcutaneous emphysema after ESD without gross perforation defects. Frank perforation was diagnosed when definite esophageal wall defect could be visualized during or after ESD. Bleeding related to ESD was defined as gastrointestinal bleeding that required further hemostatic treatment after the completion of ESD procedure and/or blood transfusion during or after the procedure. Stricture was defined as the presence of dysphagia requiring intervention.

### Statistical analysis

Therapeutic efficacy (EnR, CR, and CuR rates) was assessed per lesion. Data were presented as means ± SD or number (%). Categorical variables were compared using Chi-square test or Fisher exact test while continuous variables were compared using Student’s t test or Mann-Whitney rank sum test. Multivariate analysis was performed to evaluate whether GA was associated with better short-term outcomes. A *p* value of less than 5% was considered to be statistically significant. All statistical analyses were performed using SPSS version 23.0 (SPSS Inc., Chicago, IL, USA).

## Results

### Clinicopathologic characteristics

The clinicopathologic features of the 169 patients with 175 SESCC lesions were summarized in Table 1. The mean age was 64.5 ± 7.9 years. A total of 157 (92.9%) patients were men. Thirteen (7.4%), 46 (26.3%), 113 (64.6%), and 3 (1.7%) lesions were located in the upper-, middle-, lower-esophagus, and the gastroesophageal junction, respectively. Macroscopic type IIa, IIb, IIc, and mixed type were found in 26 (14.9%), 125 (71.4%), 21 (12.0%), and 3 (1.7%) tumors, respectively. The mean gross tumor size was 1.5 ± 0.9 cm and the mean pathologic tumor size of the resected specimen was 1.5 ± 0.8 cm. Thirty-four cancers (19.4%), 140 (80.0%), and 1 (0.6%) had well differentiated, moderately differentiated, and poorly differentiated histology, respectively. Most tumors (62.3%) had a circumferential size of < 1/2 but ≥1/4 of the lumen. Most post-ESD mucosal defect (48.6%) had a circumferential size < 3/4 but ≥1/2 of the lumen. Regarding the depth of tumor invasion, 26 (14.9%), 71 (40.6%), 42 (24.0%), 10 (5.7%), and 26 (14.9%) lesions were classified as M1, M2, M3, SM1, and SM2&3, respectively. Esophageal ESD was performed under CS for 93 (53.1%) lesions and under GA for 82 (46.9%) lesions.

**Table 1:** Clinicopathologic characteristics of 169 patients with 175 superficial esophageal squamous cell carcinomas in this study

Variables	
Age (years)	64.5 ± 7.9
Gender, male	157 (92.9)
Body mass index (Kg/m^2^**)**	23.6 ± 2.9
Smoking	
Current smoker	32 (18.9)
Ex-smoker	81 (47.9)
Never-smoker	56 (33.1)
Alcohol	
None	95 (56.2)
> 1 day/month	18 (10.7)
> 1 day/week	27 (16.0)
> 4 days/week	29 (17.2)
Diabetes mellitus (yes)	25 (14.8)
Hypertension (yes)	51 (30.2)
Tumor location	
Upper thoracic	13 (7.4)
Middle thoracic	46 (26.3)
Lower thoracic	113 (64.6)
Esophagogastric junction	3 (1.7)
Tumor morphology	
IIa	26 (14.9)
IIb	125 (71.4)
IIc	21 (12.0)
Mixed	3 (1.7)
Gross tumor size (cm)	1.5 ± 0.9
Pathologic tumor size (cm)	1.5 ± 0.8
Histologic differentiation	
Well differentiated	34 (19.4)
Moderately differentiated	140 (80.0)
Poorly differentiated	1 (0.6)
Circumferential size of the tumor (of lumen)	
< 1/4	50 (28.6)
≥ 1/4, < 1/2	109 (62.3)
≥ 1/2, < 3/4	16 (9.1)
≥ 3/4	0 (0)
Circumferential size of the post-ESD mucosal defect (of lumen)	
< 1/4	5 (2.9)
≥ 1/4, < 1/2	68 (38.9)
≥ 1/2, < 3/4	85 (48.6)
≥ 3/4	17 (9.7)
Depth of tumor invasion	
M1	26 (14.9)
M2	71 (40.6)
M3	42 (24.0)
SM1	10 (5.7)
SM2&3	26 (14.9)
Method of sedation	
Conscious sedation	93 (53.1)
General anesthesia	82 (46.9)

Data are presented as mean ± SD or number (%) of patients or lesions

### Short-term outcomes and complications

Short-term outcomes were shown in Table 2. EnR was achieved for 164 (93.7%) lesions. CR was achieved for 131 (74.9%) lesions. Forty-four lesions with non-CR (25.1%) were due to piecemeal resection (*n* = 10) and positive resection margin (*n* = 40). CuR was achieved in 103 lesions (58.9%). Seventy-two lesions with non-CuR were due to non-CR (*n* = 44), SM invasion (*n* = 36), and LVI (*n* = 20). Among the 72 patients with non-CuR, 33 underwent additional treatment: 25 received surgery; 3 received argon plasma coagulation (APC) ablation; 2 received chemo-radiation therapy; 2 received radiation therapy; and 1 received further endoscopic mucosal resection (Fig. 1). The mean procedure time was 64.4 ± 46.2 mins and the mean duration of hospital stay was 6.7 ± 4.5 days.

**Table 2:** Short-term outcomes of endoscopic submucosal dissection for superficial esophageal squamous cell carcinoma

Variables	
En bloc resection (yes)	164 (93.7)
Complete resection (yes)	131 (74.9)
Non-complete resection	44 (25.1)
Piecemeal resection	10 (22.7)
Positive total margin	40 (90.9)
Positive lateral margin	30 (68.2)
Positive vertical margin	22 (50.0)
Curative resection (yes)	103 (58.9)
Non-curative resection	72 (41.1)
Non-complete resection	44 (61.1)
SM invasion	36 (50.0)
Positive lymphatic invasion	18 (25.0)
Positive vascular invasion	2 (2.8)
Procedure time (mins)	64.4 ± 46.2
Hospital stay (days)	6.7 ± 4.5

Data are presented as mean ± SD or number (%) of patients or lesions

Procedure-related perforation occurred in 14 (8.0%) lesions. Among 4 (2.3%) patients with frank perforation, 3 (1.7%) were controlled by immediate endoscopic clipping using Quickclips (Olympus, Tokyo, Japan) and 1 (0.6%) underwent operation. Among 10 (5.7%) patients with microperforation, 4 (2.3%) received endoscopic clipping and the remaining 6 (3.4%) had only conservative management including intravenous antibiotics and fasting. No patients with microperforation underwent operation. No patients experienced post-ESD bleeding that required blood transfusion or additional endoscopy for hemostasis.

Post-ESD stricture developed in 21 (12.0%) lesions which corresponded to 21 patients. The mean tumor size of these 21 lesions was 2.1 ± 0.8 cm. Of these 21 lesions, 10 lesions (47.6%) had a post-ESD mucosal defect size larger than 3/4 of the circumference of the lumen, 9 lesions (42.9%) were between 1/2 and 3/4 and the remaining 2 lesions (9.5%) were between 1/2 and 1/4. Twenty patients (11.4%) received balloon dilatation (median 2 sessions, range 1–11 sessions). Three (1.7%) received temporary fully covered esophageal metallic stent insertion, Bonastent (Standard Sci-Tech Inc., Seoul, South Korea), after balloon dilatations (4–10 sessions). One (0.6%) received temporary esophageal stent insertion without prior balloon dilatation. Preventive managements for post-ESD stricture were applied in 18 (10.3%) lesions with large mucosal defect. Among them, polyglycolic acid sheet, NEOVEIL (Gunze, Ayabe, Japan), were applied at the ESD-induced mucosal defect for 6 (3.4%) lesions and oral prednisolone was administered in 12 (6.9%). However, among these lesions that received preventive measures, post-ESD esophageal strictures occurred in 9 (50.0%) lesions (Table 3).

**Table 3:** Complications of endoscopic submucosal dissection for superficial esophageal squamous cell carcinoma

Variables	
Perforation (yes)	14 (8.0)
Frank perforation	4 (2.3)
Clipping	3 (1.7)
Operation	1 (0.6)
Microperforation	10 (5.7)
Clipping	4 (2.3)
Conservative management	6 (3.4)
Bleeding (yes)	0 (0)
Stricture (yes)	21 (12.0)
Balloon dilatation	20 (11.4)
Balloon dilatation and temporary stent insertion	3 (1.7)
Temporary stent insertion	1 (0.6)
Preventive treatment for stricture (yes)	18 (10.3)
Polyglycolic acid sheet application	6 (33.3)
Stricture (yes)	4 (66.6)
Oral prednisolone	12 (66.6)
Stricture (yes)	5 (41.7)

Data are shown as number (%) of lesions

### Long-term outcomes

**Fig. 3:**
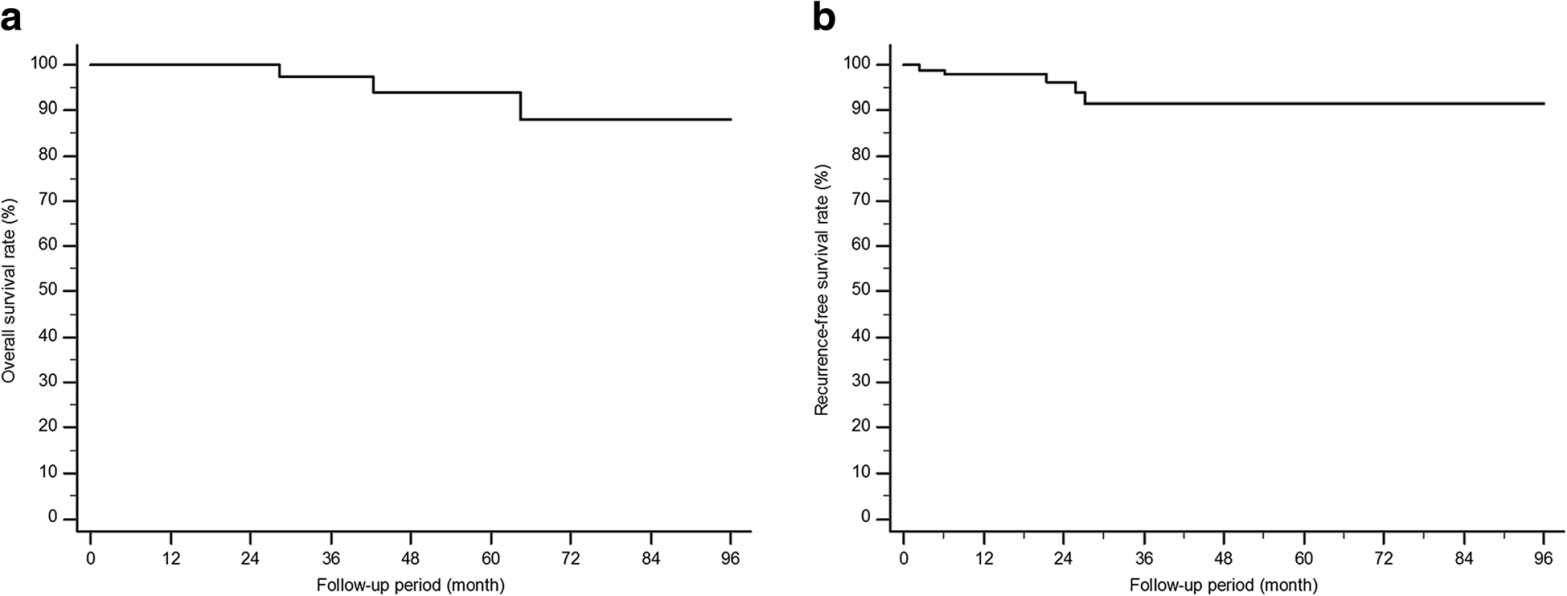
Long-term outcome analysis. **a** Overall survival in patients with curative resection. **b** Recurrence-free survival in patients with curative resection

The long-term outcomes of 99 patients achieving CuR were shown in Table 4 and Fig. 3. One-year, 3-year, and 5-year overall survival (OS) of these patients were 100, 97.3, and 93.8%, respectively. One-year, 3-year, and 5-year recurrence-free survival (RFS) of them were 97.8, 91.5, and 91.5%, respectively. Specifically, during a mean follow-up period of 33.7 months (median 22.9 months, range 2.1–109.2 months) for OS, 3 patients died without esophageal cancer recurrence. During a mean follow-up period of 32.5 months (median 22.9 months, range 2.1–109.2 months) for recurrence, 1 (1.0%) patient experienced LNM and received concurrent chemo-radiation therapy. Esophageal cancer of this patient invaded lamina propria, but there was no LVI. Resection margin was negative and the size was 1.2 ×1.3 cm. Two months after ESD, synchronous lesion close to the ESD scar was found. The patient received APC and was followed up without recurrence. However, after 18 months, CT showed a right supraclavicular lymph node enlargement and metastatic SCC was confirmed by histologic examination. Synchronous and metachronous cancers developed in 1 (1.0%) and 3 (3.0%) patients, respectively. The synchronous cancer located close to previous ESD-induced scar and was difficult to be removed by ESD. The patient denied surgery and the lesion was successfully treated with endoscopic ablation using APC. Of the 3 patients with metachronous cancers, 2 underwent repeat esophageal ESD and 1 received Ivor-Lewis operation.

**Table 4:** Long–term outcome of 99 patients achieving curative resection

Variables	
Death (yes)	3 (3.0)
Esophageal cancer-related death (yes)	0 (0.0)
Distant recurrence (yes)	1 (1.0)
Lymph node metastasis	1 (1.0)
CCRT	1 (1.0)
Local recurrence (yes)	0 (0.0)
Synchronous cancer (yes)	1 (1.0)
APC ablation	1 (1.0)
Metachronous cancer (yes)	3 (3.0)
ESD	2 (2.0)
Ivor-Lewis operation	1 (1.0)

Data are presented as number (%) of patients

*CCRT* concurrent chemo-radiation therapy, *APC* argon plasma coagulation, *ESD* endoscopic submucosal dissection

Among the 70 patients with non-CuR, 3 died without esophageal cancer recurrence.

### Clinicopathologic characteristics according to the sedation method

As shown in Table 5, there was no significant difference in terms of age, gender, and tumor location between patients/lesions receiving CS and those receiving GA. However, IIa tumor morphology (24.4% vs. 6.5%, *P* <  0.001) was more frequent, gross tumor size (1.9 ± 1.0 vs. 1.2 ± 0.6 cm, *P* <  0.001) and pathologic tumor size (1.8 ± 0.9 cm vs. 1.2 ± 0.6 cm, *P* <  0.001) were larger in the GA group than those in the CS group. In addition, large circumferential size of tumor (≥ 1/2 of lumen) and post-ESD mucosal defect (≥ 3/4 of lumen) were more frequent in the GA group than those in the CS group (13.0% vs. 3.0%, *P* = 0.005 and 17.1% vs. 3.2%, *P* <  0.001, respectively). The first 10 ESDs were more frequent in the CS group than in the GA group (40.9% vs. 15.9%, *P* <  0.001).

**Table 5:** Clinicopathologic features of patients and tumors according to the sedation method used for endoscopic submucosal dissection

Variables	Conscious sedation (*n* = 93)	General anesthesia (*n* = 82)	*p*-value
Age (years)	63.7 ± 8.0	65.3 ± 7.6	0.191
Gender, male	85 (91.4)	77 (93.9)	0.577
Tumor location			0.064
Upper thoracic	4 (4.3)	9 (11.0)	
Middle thoracic	21 (22.6)	25 (30.5)	
Lower thoracic	65 (69.9)	48 (58.5)	
Esophagogastric junction	3 (3.2)	0 (0.0)	
Tumor morphology			< 0.001
IIa	6 (6.5)	20 (24.4)	
IIb	77 (82.8)	48 (58.5)	
IIc	10 (10.8)	11 (13.4)	
Mixed	0 (0.0)	3 (3.7)	
Gross tumor size (cm)	1.2 ± 0.6	1.9 ± 1.0	< 0.001
Pathologic tumor size (cm)	1.2 ± 0.6	1.8 ± 0.9	< 0.001
Circumferential size of the tumor (of lumen)			0.005
< 1/4	24 (25.8)	26 (31.7)	
≥ 1/4, < 1/2	66 (71.0)	43 (52.4)	
≥ 1/2, < 3/4	3 (3.2)	13 (15.9)	
≥ 3/4	0 (0.0)	0 (0.0)	
Circumferential size of the post-ESD mucosal defect (of lumen)			< 0.001
< 1/4	4 (4.3)	1 (1.2)	
≥ 1/4, < 1/2	49 (52.7)	19 (23.2)	
≥ 1/2, < 3/4	37 (39.8)	48 (58.5)	
≥ 3/4	3 (3.2)	14 (17.1)	
Depth of tumor invasion			< 0.001
M1	20 (21.5)	6 (7.3)	
M2	38 (40.9)	33 (40.2)	
M3	22 (23.7)	20 (24.4)	
SM1	9 (9.7)	1 (1.2)	
SM2&3	4 (4.3)	22 (26.8)	
Experience of endoscopist, First 10 esophageal ESDs	38 (40.9)	13 (15.9)	< 0.001

Data are presented as mean ± SD or number (%) of patients or lesions

## Short-term outcomes and perforation rate according to the sedation method

Short-term outcomes and complications according to the sedation method used for ESD were shown in Table 6. EnR rate was higher in the GA group than that in the CS group (100% vs. 88.2%, *P* <  0.001). However, CR and CuR rates were not significantly different between the two groups. Procedure-related perforation occurred less in the GA group than that in the CS group (1.2% vs. 14.0%, *P* = 0.002). Procedure time was longer in the GA group than that in the CS group (75.8 ± 47.1 min vs. 52.5 ± 42.4 min, *P* = 0.001). Hospital stay was shorter in the GA group than that in the CS group (5.5 ± 1.1 days vs. 7.7 ± 5.9 days, *P* <  0.001).

**Table 6:** Short-term outcomes and complications according to the sedation method used for endoscopic submucosal dissection

Variables	Conscious sedation (*n* = 93)	General anesthesia (*n* = 82)	*p*-value
En bloc resection (yes)	82 (88.2)	82 (100)	0.001
Complete resection (yes)	64 (68.8)	67 (81.7)	0.056
Curative resection (yes)	54 (58.1)	49 (59.8)	0.878
Perforation (yes)	13 (14.0)	1 (1.2)	0.002
Frank perforation	3 (3.2)	1 (1.2)	
Microperforation	10 (10.8)	0 (0.0)	
Procedure time (mins)	52.5 ± 42.4	75.8 ± 47.1	0.001
Hospital stay (days)	7.7 ± 5.9	5.5 ± 1.1	0.001

Data are presented as mean ± SD or number (%) of patients or lesions

Multivariate analysis revealed that GA was associated with a higher CR rate and a lower perforation rate as compared to CS (odds ratio 3.401, 95% confidence interval 1.317–8.785, *P* = 0.011 and odds ratio 0.067, 95% confidence interval 0.006–0.775, *P* = 0.030, respectively, Table 7).

**Table 7:** Multivariate analysis of factors associated with En bloc resection, complete resection, and procedure-related perforation

Variables	En bloc resection		Complete resection		Perforation	
	OR (95% CI)	*p*-value	OR (95% CI)	*p*-value	OR (95% CI)	*p*-value
Age	1.009 (0.921–1.105)	0.844	0.9991 (0.944–1.041)	0.713	1.029 (0.948–1.116)	0.494
Tumor location		0.950		0.376		0.647
Upper thoracic	1		1		1	
Middle thoracic	N/A	0.998	0.822 (0.171–3.959)	0.807	0.483 (0.034–6.948)	0.592
Lower thoracic	N/A	0.998	1.203 (0.276–5.240)	0.805	0.259 (0.020–3.309)	0.298
Esophagogastric Jx	N/A	1.000	0.140 (0.007–2.640)	0.189	N/A	0.999
Tumor morphology		0.734		0.955		0.933
IIa	1		1		1	
IIb	4.775 (0.318–71.736)	0.258	0.830 (0.253–2.728)	0.759	N/A	0.998
IIc	N/A	0.998	1.063 (0.225–5.030)	0.939	N/A	0.998
Mixed	N/A	1.000	0.511 (0.024–10.810)	0.666	N/A	1.000
Gross tumor size (cm)	0.294 (0.063–1.374)	0.120	1.320 (0.630–2.768)	0.462	0.907 (0.236–3.488)	0.888
Resected specimen size (cm)	1.147 (0319–4.126)	0.834	0.475 (0.228–0.989)	0.047	1.926 (0.580–6.397)	0.285
Depth of tumor invasion		0.744		0.228		0.924
Mucosal cancer	1		1		1	
SM invasion	1.467 (0.147–14.623)		0.567 (0.226–1.427)		0.916 (0.150–5.596)	
Experience of endoscopist		0.098		0.146		0.052
First 10 esophageal ESDs	1		1		1	
Further esophageal ESDs	0.226 (0.039–1.315)		0.513 (0.208–1.262)		0.289 (0.082–1.011)	
Method of sedation		0.996		0.011		0.030
Conscious sedation	1		1		1	
General anesthesia	N/A		3.401 (1.317–8.785)		0.067 (0.006–0.775)	

*OR* odds ratio, *CI* confidence interval, *ESD* endoscopic submucosal dissection

## Discussion

Esophageal ESD has become widely accepted for the treatment of SESCC [17, 18, 23, 36, 37]. However, its indication and curative criteria have not been well established due to scanty outcome data. Thus, this study investigated the outcomes of ESD for SESCC based on a large Korean single center experience. To the best of our knowledge, this is the first study that demonstrates higher CR rate and less perforation rate in esophageal ESD under GA as compared to those under CS.

Our CuR rate (58.9%) was lower than that of the recent series [17, 18]. Park et al. [18] reported a higher CuR rate of 77.0% for esophageal ESD in treating superficial esophageal neoplasm (*n* = 261). However, their study population consisted of a significant number of dysplasia cases (70 cases, 26.8%) and less SESCCs with SM invasion cases (19 cases, 7.3%) as opposed to no dysplasia cases and higher SESCCs with SM invasion cases (36 cases, 20.6%) in our study. Similarly, in a Japanese multicenter study, CuR rate was reported to be higher at 76.2% (95% CI: 71.5–80.3%) for esophageal ESD in treating esophageal neoplasm (*n* = 368) [17]. They, too, included a significant number of dysplasia cases (111 cases, 30.2%). Furthermore, criteria for CuR was more inclusive as it was defined as a tumor within SM1 and thus, reducing the number of SESCC (23 cases, 6.3%) with SM invasion that meet the non-CuR criteria (SM2 or deeper invasion) [17].

Depth of tumor invasion is known to be associated with the risk of LNM [15, 38]. In a study assessing the accuracy of EUS for superficial esophageal cancers, the positive and negative predictive values of EUS for SM invasion were 67 and 86%, respectively [39]. According to a recent meta-analysis, the sensitivity and specificity of EUS for T1a staging (from T1b) was 0.85 (95% CI, 0.82–0.88) and 0.87 (95% CI, 0.84–0.90), respectively [40]. Although magnifying endoscopy with narrow-band imaging has also been used for evaluating depth of tumor invasion, the additional benefit to white-light imaging is questionable [41, 42]. In addition to the depth of tumor invasion, LVI could be investigated for an accurate assessment of the risk of LNM. The risk of LNM is higher in M3 cancer with LVI than that in SM1 cancer without LVI [15, 38]. In this background, esophageal ESD could be applied as a reliable evaluation modality for LNM, although it is a therapeutic procedure for SESCC. Because we performed ESD for patients with SESCC even though minute SM invasion had already been suspected during EGD and EUS examinations, many SESCC cases with SM invasion ended up being included in the current study. This might have resulted in a lower CuR rate.

In this study, there was no death from esophageal cancer recurrence in patients who underwent CuR, and 3% of patients died from other causes. In a multicenter retrospective cohort study that investigated clinical outcomes of esophageal ESD for SESCC, 0.5% died from esophageal cancer, 5.6% died from other causes. Our study’s result is comparable with this study [17]. EnR and CR rates were also similar to other study [24].

As the esophageal wall is thin and the lumen is narrow, a stable working field without rapid and/or unexpected movements rendered by GA could facilitate the performance of esophageal ESD with a decreased risk of complication. In this study, CR rate was higher and perforation rate was lower in ESD cases under GA compared to those under CS after adjusting several clinicopathologic factors including experience of endoscopist. Non-complete resection was the most common cause of non-CuR in this study. Thus, increasing CR rate by applying GA for esophageal ESD may improve oncologic outcomes in patients with SESCC. The increased cost of GA is only about US $30–40 in Korea as compared to conscious sedation. Although cost-effectiveness may be different among countries, this study could suggest that esophageal ESD under general anesthesia improves the clinical outcomes of ESD for esophageal cancer. However, although several clinicopathologic factors and experience of endoscopist were adjusted in the multivariate analysis, there is a significant time difference between ESD under CS and GA in this study with the time spent under GA being considerably longer than CS. There could be a risk of potential confounders like advancement of ESD techniques and learning curve effects which should act in favor of GA as GA was introduced in the latter part of the study. However, the ESD lesions resected under GA were considerably larger than the lesions under CS (1.9 ± 1.0 vs. 1.2 ± 0.6, *p* <  0.001). This is not surprising as endoscopist encountering large lesions would often seek GA as the preferred mode of sedation for a better working environment during ESD.

In this study, stricture was defined as the cases receiving intervention to relieve dysphagia. Although other studies also included cases where standard endoscope could not pass through the narrowed lumen, these cases usually undergo intervention. Thus, our stricture rate may not be underestimated. In this study, stricture rate was 12.0% despite the application of preventive managements for post-ESD stricture in cases with large post-ESD mucosal defect. Polyglycolic acid sheet and oral prednisolone could not reduce post-ESD stricture rate below 50% when applied to cases with a large mucosal defect. Stricture is the most common complication of esophageal ESD and its risk increases when the circumference of the mucosal defect is more than 75% of the luminal circumference [43]. Thus, development of effective strategy to reduce post-ESD stricture is imperative for a wider use of esophageal ESD. In this study, patients with stricture received median 2 sessions of balloon dilatation and/or temporary esophageal stent insertion. Considering the limited current treatment options for stricture, development of more effective prevention methods is required.

This study has some limitations. Firstly, it was a single center retrospective study. However, most data on characteristics of patients and tumors, procedure factors, and short-term ESD outcomes were obtained from a prospectively collected database which could minimize the risk of bias. Secondly, ESD under CS was performed in the earlier period of the present study while ESD under GA was in the latter. In addition to the effects of anesthesia per se, improvement of overall ESD technologies (knife, coagulator, etc.) and institutional experience, thus, might also affect the short-term outcomes of ESD with different anesthesia types. Thirdly, the follow-up period was short to demonstrate the definite long-term outcomes of ESD for SESCC. On the other hand, this study was a rather large case series. Lastly, poor outcomes of CS might contribute to statistically significant results. However, the major finding of this study is that CS is an independent risk factor for incomplete resection and perforation. Indeed, CR rate (81.7%) under GA is comparable to that (84.5%) of a recent Japanese multicenter study [17] and perforation rate (1.2%) under GA is even better than that (5.2%) of the study. Thus, GA appears to improve the outcomes of esophageal ESD.

## Conclusion

Our results suggest that ESD is a safe and effective treatment for SESCC, particularly when CuR is achieved. Applying GA for esophageal ESD could improve the clinical outcomes of ESD in patients with SESCC.

## Abbreviations

APC: Argon plasma coagulation
CR: Complete resection
CS: Conscious sedation
CT: Computed tomography
CuR: Curative resection
EGC: Early gastric cancer
EnR: En bloc reserction
ESD: Endoscopic submucosal dissection
EUS: Endoscopic ultrasound
GA: General anesthesia
LNM: Lymph node metastasis
LVI: Lymphovascular invasion
OS: Overall survival
RFS: Recurrence free survival
SCCs: Squamous cell carcinomas
SD: Standard deviation
SESCC: Superficial esophageal squamous cell carcinoma
SM: Submucosal

## References

[1] Fitzmaurice C, Dicker D, Pain A, Hamavid H, Moradi-Lakeh M, Global Burden of Disease Cancer Collaboration (2015). the global burden of Cancer 2013. JAMA Oncol.

[2] Ferlay J, Soerjomataram I, Dikshit R, Eser S, Mathers C, Rebelo M (2015). Cancer incidence and mortality worldwide: sources, methods and major patterns in GLOBOCAN 2012. Int J Cancer.

[3] Goda K, Singh R, Oda I, Omae M, Takahashi A, Koike T (2013). Current status of endoscopic diagnosis and treatment of superficial Barrett's adenocarcinoma in Asia-Pacific region. Dig Endosc.

[4] Ra J, Paulson EC, Kucharczuk J, Armstrong K, Wirtalla C, Rapaport-Kelz R (2008). Postoperative mortality after esophagectomy for cancer: development of a preoperative risk prediction model. Ann Surg Oncol.

[5] Chang AC, Ji H, Birkmeyer NJ, Orringer MB, Birkmeyer JD (2008). Outcomes after transhiatal and transthoracic esophagectomy for cancer. Ann Thorac Surg.

[6] Connors RC, Reuben BC, Neumayer LA, Bull DA (2007). Comparing outcomes after transthoracic and transhiatal esophagectomy: a 5-year prospective cohort of 17,395 patients. J Am Coll Surg.

[7] Takahashi H, Arimura Y, Masao H, Okahara S, Tanuma T, Kodaira J (2010). Endoscopic submucosal dissection is superior to conventional endoscopic resection as a curative treatment for early squamous cell carcinoma of the esophagus (with video). Gastrointest Endosc.

[8] Lee KS, Oh DK, Han MA, Lee HY, Jun JK, Choi KS (2011). Gastric cancer screening in Korea: report on the national cancer screening program in 2008. Cancer Res Treat.

[9] Shimizu Y, Takahashi M, Yoshida T, Ono S, Mabe K, Kato M (2013). Endoscopic resection (endoscopic mucosal resection/ endoscopic submucosal dissection) for superficial esophageal squamous cell carcinoma: current status of various techniques. Dig Endosc.

[10] Kumagai Y, Monma K, Kawada K (2004). Magnifying chromoendoscopy of the esophagus: in-vivo pathological diagnosis using an endocytoscopy system. Endoscopy.

[11] Yoshida T, Inoue H, Usui S, Satodate H, Fukami N, Kudo SE (2004). Narrow-band imaging system with magnifying endoscopy for superficial esophageal lesions. Gastrointest Endosc.

[12] Choi LJ, Lee NR, Kim SG, Lee WS, Park SJ, Kim JJ (2016). Short-term outcomes of endoscopic submucosal dissection in patients with early gastric Cancer: a prospective multicenter cohort study. Gut Liver..

[13] Min BH, Kim ER, Kim KM, Park CK, Lee JH, Rhee PL (2015). Surveillance strategy based on the incidence and patterns of recurrence after curative endoscopic submucosal dissection for early gastric cancer. Endoscopy.

[14] Pyo JH, Lee H, Min BH, Lee JH, Choi MG, Lee JH (2016). Long-term outcome of endoscopic resection vs. surgery for early gastric Cancer: a non-inferiority-matched cohort study. Am J Gastroenterol.

[15] Eguchi T, Nakanishi Y, Shimoda T, Iwasaki M, Igaki H, Tachimori Y (2006). Histopathological criteria for additional treatment after endoscopic mucosal resection for esophageal cancer: analysis of 464 surgically resected cases. Mod Pathol.

[16] Soetikno R, Kaltenbach T, Yeh R, Gotoda T (2005). Endoscopic mucosal resection for early cancers of the upper gastrointestinal tract. J Clin Oncol.

[17] Tsujii Y, Nishida T, Nishiyama O, Yamamoto K, Kawai N, Yamaguchi S (2015). Clinical outcomes of endoscopic submucosal dissection for superficial esophageal neoplasms: a multicenter retrospective cohort study. Endoscopy.

[18] Park HC, Kim DH, Gong EJ, Na HK, Ahn JY, Lee JH (2016). Ten-year experience of esophageal endoscopic submucosal dissection of superficial esophageal neoplasms in a single center. Korean J Intern Med.

[19] Kim GH, Jee SR, Jang JY, Shin SK, Choi KD, Lee JH (2014). Stricture occurring after endoscopic submucosal dissection for esophageal and gastric tumors. Clin Endosc..

[20] Kawaguchi G, Sasamoto R, Abe E, Ohta A, Sato H, Tanaka K (2015). The effectiveness of endoscopic submucosal dissection followed by chemoradiotherapy for superficial esophageal cancer. Radiat Oncol.

[21] Ikeda A, Hoshi N, Yoshizaki T, Fujishima Y, Ishida T, Morita Y (2015). Endoscopic submucosal dissection (ESD) with additional therapy for superficial esophageal Cancer with submucosal invasion. Intern Med.

[22] Kim DH, Jung HY, Gong EJ, Choi JY, Ahn JY, Kim MY (2015). Endoscopic and oncologic outcomes of endoscopic resection for superficial esophageal neoplasm. Gut Liver.

[23] Probst A, Aust D, Markl B, Anthuber M, Messmann H (2015). Early esophageal cancer in Europe: endoscopic treatment by endoscopic submucosal dissection. Endoscopy.

[24] Joo DC, Kim GH, Park DY, Jhi JH, Song GA (2014). Long-term outcome after endoscopic submucosal dissection in patients with superficial esophageal squamous cell carcinoma: a single-center study. Gut Liver..

[25] Park JS, Youn YH, Park JJ, Kim JH, Park H (2016). Clinical outcomes of endoscopic submucosal dissection for superficial esophageal squamous neoplasms. Clin Endosc.

[26] Yamashina T, Ishihara R, Nagai K, Matsuura N, Matsui F, Ito T (2013). Long-term outcome and metastatic risk after endoscopic resection of superficial esophageal squamous cell carcinoma. Am J Gastroenterol.

[27] Ono S, Fujishiro M, Niimi K, Goto O, Kodashima S, Yamamichi N (2009). Long-term outcomes of endoscopic submucosal dissection for superficial esophageal squamous cell neoplasms. Gastrointest Endosc.

[28] Yamashita K, Shiwaku H, Ohmiya T, Shimaoka H, Okada H, Nakashima R (2016). Efficacy and safety of endoscopic submucosal dissection under general anesthesia. World J Gastrointest Endosc.

[29] Fujishiro M, Yahagi N, Kakushima N, Kodashima S, Muraki Y, Ono S (2006). Endoscopic submucosal dissection of esophageal squamous cell neoplasms. Clin Gastroenterol Hepatol.

[30] Fujishiro M, Kodashima S, Goto O, Ono S, Niimi K, Yamamichi N (2009). Endoscopic submucosal dissection for esophageal squamous cell neoplasms. Dig Endosc.

[31] Iizuka T, Kikuchi D, Yamada A, Hoteya S, Kajiyama Y, Kaise M (2015). Polyglycolic acid sheet application to prevent esophageal stricture after endoscopic submucosal dissection for esophageal squamous cell carcinoma. Endoscopy.

[32] Kataoka M, Anzai S, Shirasaki T, Ikemiyagi H, Fujii T, Mabuchi K (2015). Efficacy of short period, low dose oral prednisolone for the prevention of stricture after circumferential endoscopic submucosal dissection (ESD) for esophageal cancer. Endosc Int Open.

[33] Wang W, Ma Z (2015). Steroid administration is effective to prevent strictures after endoscopic esophageal submucosal dissection: a network meta-analysis. Medicine (Baltimore).

[34] Berry MF (2014). Esophageal cancer: staging system and guidelines for staging and treatment. J Thorac Dis.

[35] Japan Esophageal Society (2017). Japanese classification of esophageal Cancer, 11th edition: part I. Esophagus.

[36] Pimentel-Nunes P, Dinis-Ribeiro M, Ponchon T, Repici A, Vieth M, De Ceglie A (2015). Endoscopic submucosal dissection: European Society of Gastrointestinal Endoscopy (ESGE) guideline. Endoscopy.

[37] Kuwano H, Nishimura Y, Oyama T, Kato H, Kitagawa Y, Kusano M, et al. Guidelines for diagnosis and treatment of carcinoma of the esophagus April 2012 edited by the Japan esophageal society. Esophagus 2015. 12:1–30. 10.1007/s10388-014-0465-1 PMC4297610 25620903

[38] Akutsu Y, Uesato M, Shuto K, Kono T, Hoshino I, Horibe D (2013). The overall prevalence of metastasis in T1 esophageal squamous cell carcinoma: a retrospective analysis of 295 patients. Ann Surg.

[39] Rampado S, Bocus P, Battaglia G, Ruol A, Portale G, Ancona E (2008). Endoscopic ultrasound: accuracy in staging superficial carcinomas of the esophagus. Ann Thorac Surg.

[40] Thosani N, Singh H, Kapadia A, Ochi N, Lee JH, Ajani J (2012). Diagnostic accuracy of EUS in differentiating mucosal versus submucosal invasion of superficial esophageal cancers: a systematic review and meta-analysis. Gastrointest Endosc.

[41] Ebi M, Shimura T, Yamada T, Mizushima T, Itoh K, Tsukamoto H, et al. Multicenter, prospective trial of white-light imaging alone versus white-light imaging followed by magnifying endoscopy with narrow-band imaging for the real-time imaging and diagnosis of invasion depth in superficial esophageal squamous cell carcinoma. Gastrointest Endosc 2015;81:1355–61 e2. 10.1016/j.gie.2014.11.015 25683023

[42] Singh A, Konda VJ, Siddiqui U, Xiao SY, Waxman I (2015). Use of narrow-band imaging with magnification to predict depth of invasion of early esophageal squamous cell cancer and to guide endoscopic therapy. Gastrointest Endosc.

[43] Jain D, Singhal S (2016). Esophageal stricture prevention after endoscopic submucosal dissection. Clin Endosc..

